# Using Hebbian-Type Stimulation to Rescue Arm Function After Stroke: Study Protocol for a Randomized Clinical Trial

**DOI:** 10.3389/fncir.2021.789095

**Published:** 2022-02-10

**Authors:** Rong Xu, Guang-Yue Zhu, Jun Zhu, Yong Wang, Xiang-Xin Xing, Lin-Yu Chen, Jie Li, Fu-Qiang Shen, Jian-Bing Chen, Xu-Yun Hua, Dong-Sheng Xu

**Affiliations:** ^1^Shanghai Zhaxin Traditional Chinese and Western Medicine Hospital, Shanghai, China; ^2^Shanghai Yangzhi Rehabilitation Hospital, Shanghai, China; ^3^Tongji Hospital Affiliated to Tongji University, Shanghai, China; ^4^Yueyang Hospital of Integrated Traditional Chinese and Western Medicine, Shanghai University of Traditional Chinese Medicine, Shanghai, China; ^5^School of Rehabilitation Science, Shanghai University of Traditional Chinese Medicine, Shanghai, China

**Keywords:** stroke, rehabilitation, plasticity, transcranial magnetic stimulation (TMS), primary motor cortex (M1), supplementary motor area (SMA)

## Abstract

**Background:**

Upper-extremity hemiplegia after stroke remains a significant clinical problem. The supplementary motor area (SMA) is vital to the motor recovery outcomes of chronic stroke patients. Therefore, rebuilding the descending motor tract from the SMA to the paralyzed limb is a potential approach to restoring arm motor function after stroke. Paired associative stimulation (PAS), which is based on Hebbian theory, is a potential method for reconstructing the connections in the impaired motor neural circuits. The study described in this protocol aims to assess the effects of cortico–peripheral Hebbian-type stimulation (HTS), involving PAS, for neural circuit reconstruction to rescue the paralyzed arm after stroke.

**Methods:**

The study is a 4-month double-blind randomized sham-controlled clinical trial. We will recruit 90 post-stroke individuals with mild to moderate upper limb paralysis. Based on a 1:1 ratio, the participants will be randomly assigned to the HTS and sham groups. Each participant will undergo 5-week HTS or sham stimulation. Assessments will be conducted at baseline, immediately after the 5-week treatment, and at a 3-month follow-up. The primary outcome will be the Wolf Motor Function Test (WMFT). The secondary outcomes will be Fugl-Meyer Assessment for Upper Extremity (FMA-UE), Functional Independence Measure (FIM), and functional near-infrared spectroscopy (fNIRS) parameters. The adverse events will be recorded throughout the study.

**Discussion:**

Upper-limb paralysis in stroke patients is due to neural circuit disruption, so the reconstruction of effective motor circuits is a promising treatment approach. Based on its anatomical structure and function, the SMA is thought to compensate for motor dysfunction after focal brain injury at the cortical level. Our well-designed randomized controlled trial will allow us to analyze the clinical efficacy of this novel Hebbian theory-based neuromodulation strategy regarding promoting the connection between the cortex and peripheral limb. The results may have significance for the development and implementation of effective neurorehabilitation treatments.

**Clinical Trial Registration:**

[www.ClinicalTrials.gov], identifier [ChiCTR2000039949].

## Background

The 2013 National Epidemiological Survey of Stroke (NESS) in China reported a large and increasing burden caused by stroke, with approximately 11 million prevalent cases of stroke, 2.4 million new-onset cases of stroke, and 1.1 million stroke-related deaths in China annually, which match the long-term trends (Wu et al., 2019). Upper-extremity hemiplegia after stroke remains a clinical challenge, with only 20% of patients currently recovering normal hand function (Kwakkel et al., 2003). Common rehabilitation options to improve arm motor function include physical fitness therapy, task-oriented practice, constraint-induced movement therapy, and robot-assisted therapy. However, the clinical outcomes tend to be modest, and several disabilities remain unresolved and need further research.

In the search for improved stroke treatments, scientists have been trying to identify the link between motor function recovery after stroke and cortical reorganization, mostly focusing on plastic changes in the sensory cortex (S1), primary motor cortex (M1), supplementary motor area (SMA), premotor cortex (PMC), and cerebellum (Qing and Li, 2015). The PMC and SMA, which contribute fibers to the corticospinal tract, are major cortical regions of voluntary action plan formation and initiation (Calautti et al., 2007). They are believed to play a role in the temporal control of movement and are critical in motor function recovery in stroke patients (Kantak et al., 2012). The SMA has been reported to be a key brain area for motor function recovery after left subcortical stroke (Wan-Wa et al., 2013). As a part of the internal capsule, about 10% of the corticospinal tract fibers arise from the SMA and terminate in the spinal cord (Baran et al., 2016). The motor neurons innervating the hand muscles are mostly located at the lower cervical segments (C7–T1). A retrograde tracer study by Dum and Strick (2002) showed that corticospinal efferents from the SMA in macaques largely project to these segments. In chronic stroke patients, the overlap index of the SMA is positively associated with the Motor Status Scale (MSS) forearm-specific score (Wan-Wa et al., 2013). Schulz et al. (2017) found that the residual motor function output of chronic stroke patients depends on the degree of disruption to the corticospinal tract and fibers connecting M1 and the ventral premotor cortex. Anne et al. (2011) reported that the positive coupling of the SMA and PMC with the ipsilesional M1 decreased during the acute phase of stroke. The coupling parameters between these regions increased with increased motor recovery, which can be used to predict improved outcomes (Anne et al., 2011). Therefore, rebuilding the descending motor tract from the SMA to the peripheral limb is a potential approach for arm motor function restoration after stroke.

Recently, it has become popular to use non-invasive neuromodulation combined with standard upper-limb rehabilitation for functional restoration in patients with post-stroke hemiplegia (Edwardson et al., 2013). Repetitive transcranial magnetic stimulation (rTMS) is a non-invasive treatment that rarely induces pain caused by skin impedance and has the advantage of non-invasively stimulating deep tissue areas. However, studies on the effects of TMS in stroke have mostly focused on the activation of single targets to promote focal neuron function recovery. In the 2014–2018 evidence-based guidelines on the therapeutic use of rTMS by International Federation of Clinical Neurophysiology (IFCN), several sham-controlled studies of ipsilesional high-frequency rTMS or intermittent theta-burst stimulation (iTBS) in post-stroke patients led to improvements (from marginal to significant) in balance or paretic hand motor function (Lefaucheur et al., 2020). Unfortunately, a key study on hand motor recovery during the chronic stroke stage found that 1-Hz rTMS over the contralesional M1 was not superior to sham rTMS (Harvey et al., 2018).

Our objective is to effectively restore neural circuits after stroke. Neural circuit reconstruction is similar to sensorimotor learning based on the Hebbian principles of neural plasticity. Donald Hebb Huibert et al. (2019) hypothesized that, if the activity in a presynaptic neuron is repeatedly temporally correlated with postsynaptic neuron activation, a long-lasting alteration in synaptic structure will ensue. It is highly likely that activation of a presynaptic neuron excites the postsynaptic neuron (“neurons that fire together wire together”). The Hebbian theory is widely utilized in artificial neural networks. We think that neural circuit reconstruction will be possible based on Hebbian synaptic plasticity.

Paired associative stimulation (PAS), based on Hebbian theory with a specific time interval, is an important means by which to reconstruct neural circuits. Traditionally, PAS was performed by delivering a peripheral afferent volley using electronic stimulation over the radial or median nerve prior to TMS over M1. Peripheral magnetic stimulation (PMS) can also be applied, for example to the muscles, spinal nerve roots, and peripheral nerve fibers. PMS can directly recruit 1A afferent fibers or indirectly provide strong proprioceptive inflow related to muscle contraction or the change in a joint angle, while peripheral electronic stimulation generates a significant cutaneous inflow in the afferent fibers of the lemniscus and spino-thalamic tract. Unlike peripheral electronic stimulation, PMS is painless and well tolerated (Beaulieu and Schneider, 2015). A study of 16 healthy participants revealed that PMS could modulate cortical excitability (Sato et al., 2016). Krewer et al. (2014) investigated the effect of repetitive PMS on upper-limb muscular spasm caused by stroke or brain trauma. They reported short-term effects on spasticity for wrist flexors and long-term effects on spasticity for elbow extensors (Krewer et al., 2014). Another group investigated the effect of integrated PMS and TMS in healthy participants. They found that PAS can upregulate corticospinal excitability and downregulate intracortical inhibition (Sato et al., 2016).

We aim to use PAS to rebuild a neural circuit involving SMA → internal capsule → periphery in order to restore motor function. We will perform timely coupled stimulation of presynaptic and postsynaptic neurons to restore the motor circuits instead of simply upregulating excitation in the lesion area. If HTS can be induced non-invasively by magnetically stimulating the peripheral nerve after magnetically stimulating the SMA in the human brain, this method may provide a neuromodulation approach to rebuild the motor network after stroke. We hypothesize that PMS of the peripheral nerve tens of milliseconds after TMS of the SMA related to the functioning of the upper extremities will induce spike timing-dependent plasticity. This will increase targeted cortical excitability and help to reconstruct sensorimotor pathway conduction to facilitate arm motor function recovery.

## Objectives

The main objectives of this clinical trial are to (1) investigate whether HTS can promote arm motor function recovery in chronic stroke and (2) explore the underlying mechanisms in the brain after HTS using functional near-infrared spectroscopy (fNIRS).

## Trial Design

This protocol has been designed according to the Standard Protocol Items: Recommendations for Interventional Trials (SPIRIT) guidelines. It is registered with the Chinese Clinical Trial Registry. Based on a 1:1 ratio, 90 participants will be randomly assigned to the HTS and sham groups. The prospective, single-center, double-blind, randomized, sham-controlled clinical trial will study the therapeutic effects of HTS vs. sham intervention. Each patient will undergo 1 session of real/sham HTS per day, always followed by general rehabilitation therapy, 5 days per week, with a total of 25 sessions. Three assessments will be performed in both groups: at baseline, immediately after the 5-week treatment, and at a 3-month follow-up. The effects will be measured using a variety of rating scales. The primary outcome will be the Wolf Motor Function Test (WMFT). The secondary outcomes will be the Fugl-Meyer Assessment for Upper Extremity (FMA-UE), Functional Independence Measure (FIM), and fNIRS parameters. To strengthen treatment compliance and minimize dropout, a doctor will contact the participants regularly.

## Recruitment and Sample Selection

Participants will be recruited from Shanghai Yangzhi Rehabilitation Hospital affiliated with Tongji University. Recruitment began on 11 Nov 2020 and will continue until the required sample size has been achieved. The volunteers will be carefully screened based on the inclusion and exclusion criteria. All participants, who will be given verbal and written details on the study purpose and process, will sign a written informed consent form.

## Stroke Diagnosis

Stroke will be diagnosed based on the World Health Organization definition in 1970, “stroke is rapidly developing clinical signs of focal (or global) disturbance of cerebral function, with symptoms lasting 24 h or longer, or leading to death, with no apparent cause other than of vascular origin” (Graeme, 2017).

## Eligibility Criteria

The inclusion criteria are as follows:

(1) Having a first unilateral supratentorial, ischemic stroke, and having had a stroke in 4–12 month;

(2) Upper extremity dysfunction, with FMA-UE motor score of 30–60 out of 66 (Fugl-Meyer et al., 1975);

(3) Aged 18–80 years, regardless of sex;

(4) Mini Mental State Examination (MMSE) score > 24;

(5) Written informed consent;

(6) Right handedness.

The exclusion criteria (based on the TMS safety criteria proposed by Wassermann, 1998) are as follows:

(1) Metal implant device in the head, neck, or stimulation area;

(2) Medical implant device (cardiac pacemaker or cochlea implant);

(3) Pregnancy;

(4) Current or history of epilepsy;

(5) Current or history of medications known to affect central nervous system excitability;

(6) Intracranial hypertension;

(7) Unstable fractures or joint contracture.

(8) Taking drugs that may increase the risk of epilepsy or reduce cortical excitability.

## Randomization

The participants will be randomly assigned (using computer-generated random numbers) to one of the two groups in a 1:1 ratio after baseline assessment. A research assistant (who will not be involved in eligibility screening, the intervention, outcome assessment, or data analysis) will independently conduct the randomization process following allocation concealment. The allocation sequence will be concealed from the therapists, outcome assessors, data analysts, participants, and participants’ relatives.

## Sample Size Estimation

The calculation of the sample size was based on data from previous research and our per experiment (Lai et al., 2015). As the study involves two groups, the following sample size calculation for a two-sample mean comparison was used:


$$n_{c}=\frac{{\left(Z_{1-\alpha }+Z_{1-\beta }\right)}^{2}\,\sigma^{2}\,\left(1+\frac{1}{K}\right)}{{\left(\mu_{T}-\mu_{C}-△\right)}^{2}}$$

where, Z_1–_*_α_* = 1.960, Z_1–_*_β_* = 0.842, μ_*T*_=6.6 (predicted mean in HTS group); μ_*C*_=2.3 (predicted mean in sham group), σ = 2.0 (standard deviation), *K* = 1 (ratio of participants in the HTS and sham groups), Δ = 3 (optimality bounds), α = 0.025 (2.5% one-tailed significance level), and β = 0.20 (80% power). The calculated required sample size was 37 participants per group. Assuming a 20% dropout rate, 45 participants per group (a total of 90 participants) need to be recruited.

## Intervention Groups

The HTS intervention group will undergo HTS with conventional rehabilitation. The control group will undergo sham HTS with conventional rehabilitation.

### Blinding

The allocation sequence will be placed in sealed opaque envelopes kept in a location with restricted access. The envelopes will then be delivered to the researcher responsible for implementing the intervention the day prior to the beginning of the intervention. This process will be completed independently and the allocation sequence will be concealed from the therapists, outcome assessors, data analysts, participants, and participants’ relatives. The patients and their relatives will also be blinded to group allocation. Each participant will be identified with a particular number (rather than their name), which will be used by the outcome assessor. A blinded independent data analyst (who will not be involved in recruitment, eligibility screening, intervention delivery, or outcome assessment) will conduct the data analysis.

To ensure blinding, we will follow the sham stimulation schemes used in related studies the control group. For the sham stimulation, we will use sham coils, which are similar to real coils in terms of appearance, sound, and feeling (Ruohonen et al., 2000). The sham stimulation will be performed with the same HTS procedure (without stimulation) at the same location. The parameters on the equipment display will be identical in the HTS and sham groups.

## Intervention

### Supplementary Motor Area–Erb’s Point Conduction Time

The conduction time from the SMA to Erb’s point is defined as the latency between the motor evoked potential of the first dorsal interosseous muscles induced by SMA stimulation and the compound motor action potential evoked by Erb’s point stimulation. The conduction time will be used as the inter-stimulus interval in PAS.

### Patient Posture

The patients will be seated in a comfortable armchair, and will be asked to relax with their forearms placed on the armrest in a comfortable position. They will be awake during the whole intervention.

### Transcranial Magnetic Stimulation Parameters

TMS will be applied over the ipsilesional SMA using a figure-of-eight coil with 70-mm wing diameter and a Magstim Rapid 2 stimulator (Magstim Co., Ltd., Whitland, United Kingdom) with a stimulus intensity of 120% of the resting motor threshold (RMT). The RMT is defined as the lowest stimulus intensity (with ≥ 50 μV peak-to-valley amplitude) that is required to induce a motor evoked potential in at least 5 out of 10 stimulations. A neuronavigation system (Brainsight TMS, Rogue Research Inc., Montréal, Canada) will be used to precisely position the coil over the SMA sites, with the anatomical references being obtained from on individual T1-weighted magnetic resonance images. The SMA is located at the posterior part of the superior frontal gyrus and is bordered inferolaterally by the superior frontal sulcus and posteriorly by the precentral sulcus (Brodmann area 6).

### Peripheral Magnetic Stimulation Parameters

PMS will be applied over Erb’s point on the side with hemiparesis, with the forearms placed on a stable armrest in a neutral posture. The stimulation will be delivered by a 9-cm-diameter round-shaped coil. The patient’s neck will be flexed to the opposite side, about 20–30 degrees. The coil’s handle will be placed parallel to the brachial plexus. The intensity will be adjusted to induce a just-visible contraction of the abductor digiti minimi muscle (Evelyne et al., 2007).

### Hebbian-Type Stimulation/Paired Associative Stimulation Parameters

**FIGURE 1:**
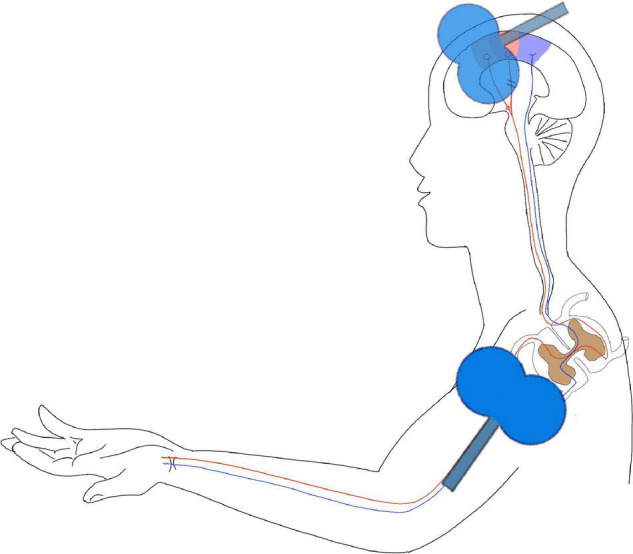
Hebbian-type stimulation (HTS). This figure is a schema chart of the intervention model of the study. In the figure, the purple area on the head is the primary sensory area, the red area is the primary motor cortex, and the position of the coil is the SMA area. In this study, the joint stimulation of the central and peripheral was used to reconstruct the pathway from the SMA area to the peripheral nerve.

The PMS pulse will be delivered prior to the TMS pulse, with an inter-stimulus interval equal to the SMA–Erb’s point conduction time, which will be determined in advance (defined as the latency between the motor evoked potential of the first dorsal interosseous muscles induced by SMA stimulation and the compound motor action potential evoked by Erb’s point stimulation). Stimuli will be delivered at 0.2 Hz, with 100 pairs, lasting for approximately 8.3 min (Figure 1).

### Intervention Stimulation

The intervention will be conducted once a day, 5 days per week, for 25 sessions in total.

### Sham Stimulation

For the sham stimulation, we will place the sham coils in the same position as in SMA TCM and PMS. The strength and frequency of the sham coil clicking noise will be similar to the real clicking noise (but with no magnetic stimulation).

### Basic Stroke Management

Each participant in both groups will receive basic stroke management, including (1) assessments at the level of body function/structure, activities and participation and environmental Factors described in the International Classification of Functioning, Disability, and Health (ICF) (Svestková, 2008), (2) rehabilitation services (delivered by a multidisciplinary team of neurologists, rehabilitation physicians, rehabilitation nurses, occupational therapists, physical therapists, and speech and language therapists) based on the recommendations of clinical guidelines for patients with stroke (Winstein et al., 2016), and (3) medications(e.g., antihypertensive drugs, anti-platelet medicine, hypoglycemia agents, anti-spasm drugs etc.).

## Assessments

The clinical assessments will be performed at baseline (on the day of enrollment), immediately after the 5-week HTS/sham intervention, and at the 3-month follow-up after the intervention. After recruitment and eligibility screening, the following data will be collected: sociodemographic data (date of birth, gender, laterality, marital status, and occupational status), neuroimaging results, disease and current episode duration, blood pressure, comorbidities (psychiatric, addiction, and somatic diseases), medications, and degree of prior resistance to other treatments. These variables will be evaluated at baseline, immediately after the 5-week HTS/sham intervention, and at the 3-month follow-up (along with WMFT, FMA-UE, FIM, and fNIRS) (Table 1).

**TABLE 1:** Study timeline.

	Enrolment	Baseline assessment	Intervention phase		Post-treatment assessment	Follow-up assessment
Time point	−T_1w_	T_0_	T_0_	T_5w_	T_5w_	T_3months_
Enrolment						
Eligibility screening	×					
Informed consent	×					
Allocation	×					
Interventions						
Hebbian-type stimulation (HTS)			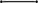			
Sham stimulation			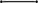			
Primary outcome						
Wolf Motor Function Test (WMFT)		×			×	×
Secondary outcomes						
Fugl-Meyer Assessment (FMA)		×			×	×
Functional Independence Measure (FAM)		×			×	×
Functional near-infrared spectroscopy (fNIRS) parameters		×			×	

## Adverse Events and Safety Considerations

Data on all potential AEs will be obtained from the participants and recorded in an observation table by the researchers (including time and date of occurrence, severity, treatment parameters, and potential causal relationship with the intervention). Monitoring of AEs will be carried out by the treating physical therapists. The principal researcher (MEAF) and data security management members will immediately be informed about severe AEs. AEs might include transient upper limb weakness, muscle soreness in the stimulation area, headache, increased muscle tone in the upper extremities, and hearing loss (caused by the coil noise during stimulation). We will give the patients earplugs to prevent hearing impairment during stimulation, and when the treatment is over, we will conduct a hearing assessment for all patients who report aural fullness, hearing loss, or tinnitus. The most serious acute AEs of TMS is epileptic seizure. Patients will be carefully asked if they have a disease that may cause epilepsy or are taking drugs that may increase the risk of epilepsy or reduce cortical excitability, and those who do will be excluded prior to randomization. If epileptic seizure occurs, the stimulation will be stopped and acute treatment will immediately be provided (Rossi et al., 2021).

## Maximizing Treatment Compliance and Minimizing Dropout Rate

To strengthen treatment compliance and minimize dropout, a doctor will contact the participants regularly by phone to confirm the appointments, assess the effect of the treatment, and discuss the subsequent treatment and any issues that may interfere with compliance.

### Primary Outcomes

WMFT is highly recommended to assess the motor ability of patients with moderate to severe upper extremity motor deficits in research and clinical settings (Wolf et al., 1989). Its test-retest and inter-rater reliability and internal consistency range from 0.88 to 0.98, with most of the values being close to 0.95 (Morris et al., 2001). It will be used to assess upper limb movement ability, encompassing 17 upper-extremity motor tasks that involve starting position and verbal instructions (Wolf et al., 2001). The Functional Ability Scale score for each task ranges from 0 to 5. It is an effective and sensitive method to assess the upper extremity functional ability of patients with moderate to severe impairments after stroke (Hodics et al., 2012).

### Secondary Outcomes

We will use fNIRS to assess the M1, S1, PMC, and SMA activities during six cycles of 15 s of hand grasping and 45 s of rest while sitting on a reclining chair. Each participant will be assessed at three time points (at baseline, immediately after the 5-week treatment, and at the 3-month follow-up). fNIRS parameters will be assessed and correlations with the clinical outcomes will be assessed. Other secondary outcomes will include activities of daily living, which will be determined using the FIM, and the motor section of FMA-UE will also be used to estimate upper limb movement ability (based on normal reflex activity and volitional movement of the upper extremities, along with the wrist, hand, coordination/speed, sensation, and passive joint motion domains). These stroke-associated measures will be assessed in addition to the primary outcome.

### Statistical Analysis

Statistical analysis will be performed using SPSS 22.0 (SPSS Inc., Chicago, IL, United States). Database management and statistical analysis will be performed by an independent researcher, who will be blinded to group allocation. Monitoring of AEs will be carried out by the treating physical therapists. The primary analysis, which will be a per-protocol analysis, will include the patients who underwent the treatment course and completed the 3-month follow-up.

**FIGURE 2:**
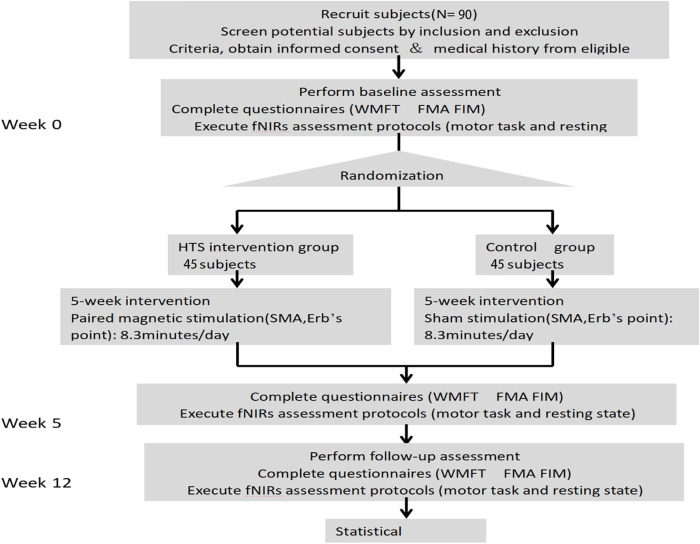
Overview of study design.

Descriptive statistics will be used to describe the baseline characteristics of the participants in both groups. The normally distributed continuous data will be presented as mean ± standard deviation, and the non-normally distributed continuous data will be presented as median (maximum and minimum). The Shapiro–Wilk test will be used to evaluate data normality. Homogeneity of variance will be assessed by Levene’s test. For the normally distributed continuous data (including WMFT, UE-FMA, and FIM), paired *t*-tests will be used to compare between baseline and postintervention in each group. Additionally, unpaired *t*-tests will be used to assess the differences in the baseline characteristics and the primary and secondary outcomes between the HTS and sham groups. For the non-normally distributed continuous data, Wilcoxon tests will be used. The one-tailed significance level (α) for statistical hypothesis testing will be set at *P* < 0.025 (Figure 2).

## Discussion

Limb motor function is controlled by neural networks in several brain regions, including the default mode network and the sensory and motor networks. The motor network includes M1, pyramidal system, supplementary motor cortex (SMA)/PMC, parietal temporal cortex, basal ganglia, and cerebellum. Sensory information is integrated through the parietal temporal cortex and then connected to the brain motor neural circuits *via* a special network. Both human and animal studies have shown that the brain areas surrounding stroke lesions can promote motor function recovery (Payne and Lomber, 2001). The non-injured areas around the lateral hemisphere after stroke have a certain degree of plasticity, likely due to the reorganization of sensorimotor networks.

This plasticity may depend on the functional overlapping regions in the sensorimotor network, which may compensate for the lost function after stroke. A study suggested a surrogate role for the SMA after M1 injury in monkeys. Before M1 injury, SMA neurons exhibited activity during learning (push-button task), which disappeared when performing the task after the task had been learnt. However, M1 injury led to SMA activity being restored when the task was performed again (Williams et al., 2006). This phenomenon is consistent with the ipsilesional SMA activation in stroke patients during rehabilitation (Hara, 2015). Zhang et al. (2016) found that compare to the health control functional connections between M1 and the ipsilesional parietal cortex, frontal gyrus, and SMA were increased in chronic stroke patients, while functional connections between bilateral M1 were decreased in stroke. Inman et al. (2012) found that the connections between the superior parietal cortex and M1, and the superior parietal cortex and the SMA, were significantly reduced in chronic stroke. The study of Jingchun et al. (2020) used fine map of fMRI showed that he CST originated from SMA are significantly related with motor outcomes in chronic stroke patients. The SMA/PMC is the key area for motor plan formation and movement initiation. The SMA/PMC and the prefrontal cortex form the brain’s cognitive network, which predicts the movements needed for tasks to be performed and receives information from the brain’s sensory integration network about the body’s spatial location, generating a primary task plan. Our study aims to restore the motor circuits, based on the ideas of Donald Hebb. He hypothesized that when the axon of cell A repeatedly or continuously participates in stimulating cell B, the synaptic connection between cell A and cell B will be strengthened. On this basis, Muming Poo found that long-term potentiation or depression can be produced by exciting the postsynaptic membrane 20 ms after or before exciting the presynaptic membrane, respectively, which is known as spike timing-dependent plasticity. The difference between spike timing-dependent plasticity and Hebbian theory is that the former emphasizes the role of the sequence of presynaptic and postsynaptic membrane excitation in synaptic remodeling (Zuzanna et al., 2019).

There is a lack of guidelines on PAS HTS for stroke rehabilitation in the subacute and chronic phases. Our planned study may complement the existing stroke rehabilitation guidelines, add to the experience in this area, and provide a basis for treatment application.

## Confidentiality

The participants’ personal data will be kept confidential. All human sample data (functional assessment results and functional imaging examinations) will be identified by study number rather than name. Identifiable information will not be disclosed to members outside the study group unless permission is obtained from the subject. All study members and study sponsors were required to keep the identity of the subjects confidential. The subjects’ files will be kept in a locked filing cabinet for researchers’ reference only.

## Trial Status

Protocol version number and date: version 1, dated February 21, 2021.

Date recruitment began: December 1, 2020.

Date when recruitment will be completed: March 3, 2022.

## Data Management

Two investigators will run the database which has been established in the clinical research database of Shanghai YangZhi Rehabilitation Hospital (Shanghai Sunshine Rehabilitation Center). One of them will enter data into the database, and the other researcher will be in charge of checking data accuracy. During the collection process, data will be stored in a password-protected online database accessible only to the researcher. Once the collections are completed, data will be kept in a password-protected file on the researcher’s personal computer.

## Coordinating Center, Steering Committee, Endpoint Adjudication Committee

No coordinating center, steering committee, endpoint adjudication committee were constituted. The investigators will hold a monthly meeting to find out any final difficulties or mistakes. The researchers met with the intervention group every 4 months to identify and correct any final difficulties in the recruitment and follow-up process, data management, monitoring, and statistical analysis of results. They are responsible for assessing the rate of progress to ensure that the trial is carried out in accordance with the research plan. The main researchers supervised the correct development of the experiment. If any unexpected adverse reactions occur, an audit process will be conducted to identify and correct the side effects. The three therapist candidates in the study will be in charge of the evaluation. They are all experienced therapists. And all the measurements will be duplicated at least 2 times to promote data quality. The main investigators have completed the online training courses of Good Clinical Practice of Pharmaceutical Products (GCP) and got the GCP certifications.

## Data Availability Statement

The original contributions presented in the study are included in the article/Supplementary Material, further inquiries can be directed to the corresponding author/s.

## Ethics Statement

The studies involving human participants were reviewed and approved by the Ethics Review Board of Shanghai Yangzhi Rehabilitation Hospital Affiliated to Tongji University. The patients/participants provided their written informed consent to participate in this study. Written informed consent was obtained from the individual(s) for the publication of any potentially identifiable images or data included in this article.

## Author Contributions

RX, JZ, J-BC, and F-QS were doctoral fellows in the project, contributed to the study design, carry out recruitment and screening of participants, set up the intervention and collect baseline data, and perform statistical analysis and interpretation of results. G-YZ and X-XX were senior researchers and carry out the intervention. YW was the occupational therapist. JL and L-YC were the physical therapists, designed the process evaluation, and performed the evaluation of cognitive and hand function. X-YH and D-SX were the chief investigators and supervisors of the project, designed the study, partake in analyzing, and interpreting the results. All authors helped draft the manuscript and consent to publication, and read and approved the final manuscript.

## Conflict of Interest

The authors declare that the research was conducted in the absence of any commercial or financial relationships that could be construed as a potential conflict of interest.

## Publisher’s Note

All claims expressed in this article are solely those of the authors and do not necessarily represent those of their affiliated organizations, or those of the publisher, the editors and the reviewers. Any product that may be evaluated in this article, or claim that may be made by its manufacturer, is not guaranteed or endorsed by the publisher.

## Abbreviations

SMA: Supplementary motor area
FMA-UE: Fugl-Meyer Assessment for Upper Extremity
WMFT: Wolf Motor Function Test
TMS: Transcranial magnetic stimulation
PAS: Paired associative stimulation
HTS: Hebbian-type stimulation
FIM: Functional Independence Measure
fNIRS: Functional near-infrared spectroscopy
M1: Primary motor cortex
NESS: National Epidemiological Survey of Stroke
PMC: Premotor cortex
rTMS: Repetitive transcranial magnetic stimulation
iTBS: Intermittent theta-burst stimulation
PMS: Peripheral magnetic stimulation
MMSE: Mini Mental State Examination
ICF: Classification of Functioning, Disability and Health
SPIRIT: Standard Protocol Items: Recommendations for Interventional Trials
AEs: Adverse events
GCP: Good Clinical Practice of Pharmaceutical Products
IFCN: International Federation of Clinical Neurophysiology.
